# Spontaneously hypertensive rats can become hydrocephalic despite undisturbed secretion and drainage of cerebrospinal fluid

**DOI:** 10.1186/s12987-023-00448-x

**Published:** 2023-07-04

**Authors:** Sara Diana Lolansen, Dagne Barbuskaite, Fenghui Ye, Jianming Xiang, Richard F. Keep, Nanna MacAulay

**Affiliations:** 1 Department of Neuroscience, University of Copenhagen, Blegdamsvej 3B, Copenhagen, DK-2200 Denmark; 2 Department of Neurosurgery, University of Michigan, Ann Arbor, USA

**Keywords:** Spontaneously hypertensive rat, Choroid plexus, Cerebrospinal fluid, Intracranial pressure, Outflow resistance, Ventriculomegaly, Hydrocephalus

## Abstract

**Background:**

Hydrocephalus constitutes a complex neurological condition of heterogeneous origin characterized by excessive cerebrospinal fluid (CSF) accumulation within the brain ventricles. The condition may dangerously elevate the intracranial pressure (ICP) and cause severe neurological impairments. Pharmacotherapies are currently unavailable and treatment options remain limited to surgical CSF diversion, which follows from our incomplete understanding of the hydrocephalus pathogenesis. Here, we aimed to elucidate the molecular mechanisms underlying development of hydrocephalus in spontaneously hypertensive rats (SHRs), which develop non-obstructive hydrocephalus without the need for surgical induction.

**Methods:**

Magnetic resonance imaging was employed to delineate brain and CSF volumes in SHRs and control Wistar-Kyoto (WKY) rats. Brain water content was determined from wet and dry brain weights. CSF dynamics related to hydrocephalus formation in SHRs were explored in vivo by quantifying CSF production rates, ICP, and CSF outflow resistance. Associated choroid plexus alterations were elucidated with immunofluorescence, western blotting, and through use of an ex vivo radio-isotope flux assay.

**Results:**

SHRs displayed brain water accumulation and enlarged lateral ventricles, in part compensated for by a smaller brain volume. The SHR choroid plexus demonstrated increased phosphorylation of the Na^+^/K^+^/2Cl^−^ cotransporter NKCC1, a key contributor to choroid plexus CSF secretion. However, neither CSF production rate, ICP, nor CSF outflow resistance appeared elevated in SHRs when compared to WKY rats.

**Conclusion:**

Hydrocephalus development in SHRs does not associate with elevated ICP and does not require increased CSF secretion or inefficient CSF drainage. SHR hydrocephalus thus represents a type of hydrocephalus that is not life threatening and that occurs by unknown disturbances to the CSF dynamics.

**Supplementary Information:**

The online version contains supplementary material available at 10.1186/s12987-023-00448-x.

## Introduction

Hydrocephalus encompasses a heterogeneous group of neurological conditions characterized by an abnormal accumulation of cerebrospinal fluid (CSF) within the cerebral ventricles. It can occur in presence or absence of changes in intracranial pressure (ICP) [1]. Hydrocephalus is associated with poor clinical outcome [2, 3] and may be fatal if left untreated [4]. The pathogenesis of hydrocephalus remains poorly understood, in part due to the limited availability of animal hydrocephalus models that do not require surgical manipulation [5]. Spontaneously hypertensive rats (SHRs) were originally created by selective inbreeding of Wistar rats with spontaneous high blood pressure to advance hypertension research [6, 7]. In addition to genetically induced hypertension, SHRs display progressive ventricular enlargement in comparison to their normotensive control counterparts, the Wistar-Kyoto (WKY) rats [8, 9], which manifests around seven weeks of age [10, 11]. The ventricular enlargement in SHRs appears not to arise as a direct consequence of the concomitant elevation in blood pressure, as pharmacological blood pressure reduction fails to attenuate the observed ventricular enlargement in SHRs and experimentally induced hypertension is insufficient to cause ventricular enlargement in naïve rats [9]. Apart from ventricular enlargement, SHRs present with smaller intracranial and brain volumes [12–14], larger ventricular and total CSF volumes [8, 11–14], and abnormal CSF flow properties [12] similar to those reported in some patients with hydrocephalus [15]. Hydrocephalus development in SHRs may thus originate from abnormal CSF dynamics. However, at present, the CSF dynamics in SHRs remain poorly characterized. Hence, whether development of hydrocephalus in SHRs is associated with pathological alterations such as an increased CSF secretion and/or an elevated CSF outflow resistance await determination. Both situations may promote accumulation of CSF and ultimately result in development of hydrocephalus if not properly compensated. Increased CSF secretion is indeed sufficient to promote hydrocephalus development as evident from humans with pathological alterations in the CSF-producing tissue, the choroid plexus [16–18], and in animals with experimentally induced posthemorrhagic hydrocephalus [19–23] and postinfectious hydrocephalus [19]. Conversely, hydrocephalus development in humans [24] and experimental animals [25, 26] has also been linked to an elevated CSF outflow resistance, reflecting inefficient CSF drainage. The pathogenesis of hydrocephalus is thus complex and likely involves an intricate interplay of various pathological mechanisms, some of which may be directly linked to abnormal CSF dynamics.

The present study, therefore, sought to elucidate whether development of hydrocephalus in SHRs is associated with abnormal CSF dynamics such as an increased CSF secretion or an elevated CSF outflow resistance. Elucidation of the molecular mechanisms underlying hydrocephalus formation in SHRs and delineation of the associated CSF dynamics may provide novel insight into the pathogenesis of hydrocephalus.

## Methods

### Animal experiments

Nine-week-old male SHRs and WKY rats (Janvier Labs, France, or Charles River Laboratories, MI, USA) were used in the study. Animals were housed in a temperature-controlled room with a 12 h:12 h light-dark cycle and had free access to a standard rodent pellet diet and tap water. Animal experiments performed at University of Copenhagen conformed to the legislations for animal protection and care in the European Community Council Directive (2010/63/EU) and were approved by the Danish Animal Experiments Inspectorate (License no. 2018-15-0201-01595). Animal experiments performed at University of Michigan were approved by the University of Michigan Committee on the Use and Care of Animals and followed the Guide for The Care and Use of Laboratory Animals (National Research Council, USA). The majority of the experiments were conducted with six rats of each type for each experimental paradigm (ICP, resistance to CSF outflow, CSF secretion measurements, immunohistochemistry), whereas five rats of each kind (based on earlier results) were deemed sufficient for magnetic resonance imaging, brain water quantification, and isotope flux experiments. For Western blotting, only four rats of each type were available for the experiments.

### Solutions

In vivo experiments were conducted with heated (37°C) and gas-equilibrated artificial CSF (aCSF; (in mM) 127 NaCl, 2.5 KCl, 2.5 CaCl_2_, 1.3 MgSO_4_, 1 NaH_2_PO_4_, 10 glucose, 25 NaHCO_3_, pH adjusted with 95% O_2_ and 5% CO_2_). Ex vivo experiments (^86^Rb^+^ efflux assays) were conducted in HEPES-containing aCSF (HEPES-aCSF; (in mM) 120 NaCl, 2.5 KCl, 2.5 CaCl_2_, 1.3 MgSO_4_, 1 NaH_2_PO_4_, 10 glucose, 17 Na-HEPES, adjusted to pH 7.4 with NaOH/HCl). HEPES-aCSF was selected, as continuous gas-equilibration of the HCO_3_^−^ buffered aCSF is technically challenging with small quantities of isotope-containing test solutions, and earlier experiments revealed similar ^86^Rb^+^ efflux rates whether the experiments were conducted in heated and gas-equilibrated aCSF or HEPES-aCSF [20].

### Anesthesia and physiological parameters

Xylazine and ketamine anesthesia was used for the surgeries (ScanVet, 10 mg/kg xylazine, 5 min later, 100 mg/kg ketamine, half dose of ketamine was re-dosed every 10–40 min upon detection of foot reflex). Animal body temperature was maintained at 37 °C by a homeothermic monitoring system (Harvard Apparatus). Rats were tracheostomized and mechanically ventilated with the VentElite system (Harvard Apparatus), inhaling 0.9 l min^− 1^ humidified air mixed with 0.1 l min^− 1^ O_2_. The ventilation was adjusted to result in 4.5 ± 0.5 kPa blood pCO_2_ according to exhaled end tidal CO_2_ measured with a capnograph (Type 340, Harvard Apparatus). Blood samples were collected from anesthetized and mechanically ventilated rats through insertion of a femoral artery catheter during the ICP measurements (described below). The blood gas content was determined with an ABL80 blood gas analyzer (Radiometer).

### Brain water quantification

The rat brain was isolated immediately following decapitation, and the wet brain weight determined on a pre-weighed porcelain evaporation beaker. The brain was afterwards placed in a pre-heated oven at 100 °C and left to dry for 3 days to a constant mass. The dry brain was weighed, and the brain water content determined using the equation: (wet weight - dry weight)/dry weight.

### Magnetic resonance imaging (MRI)

Anesthetized rats underwent MRI in a 9.4 Tesla preclinical horizontal bore scanner (BioSpec 94/30 USR, Bruker BioSpin, Ettlingen, Germany) equipped with a 240 mT/m gradient coil (BGA-12 S, Bruker) at the Preclinical MRI Core Facility, University of Copenhagen. During the scan, anesthesia was maintained at 1-1.5% isoflurane in a 1/1 mixture of air/oxygen. The MR scanner was interfaced to a Bruker Avance III console and controlled by Paravision 6.1 software (Bruker). MRI was performed with an 86 mm-inner-diameter volume resonator and a 4-channel surface quadrature array receiver coil. Animal body temperature was maintained at 37 ± 0.5 °C with a thermostatically controlled waterbed and the respiratory rate closely monitored with an MR compatible monitoring system (SA Instruments, NY, USA). The MRI protocol consisted of T_2_-weighted 2D rapid acquisition with relaxation enhancement (2D-RARE) for reference spatial planning. The following settings were applied: repetition time (TR) = 10,000 ms, effective echo time (TE) = 60 ms, number of averaging (NA) = 3, RareFactor = 16, slice thickness = 500 μm, in-plane resolution = 137 × 137 μm, 50 coronal slices, total acquisition time (TA) = 8 min. For high resolution CSF volumetry, a 3D constructive interference steady-state sequence (3D-CISS) [22, 27] image was calculated as a maximum intensity projection (MIP) from 4 realigned 3D-TrueFISP volumes with 4 orthogonal phase encoding directions (TR = 4.6 ms, TE = 2.3 ms, NA = 1, Repetitions = 2, Flip angle = 50º, 3D spatial resolution 100 × 100 × 100 μm, RF phase advance 0, 180, 90, 270º, TA = 28 min). To obtain optimal spatial uniformity, all acquired 3D-TrueFISP volumes were motion-corrected before calculation as MIP, and the image bias field was removed with Advanced Normalization Tools (ANTs) [28, 29]. For each animal, the total brain volume was automatically segmented using region growing with ITK-snap (version 4.0.0) [30]. The pixel intensity was factorized by semi-automatic thresholding to segment the lateral ventricle in each hemisphere, the third ventricle, and the fourth ventricle. Volume measurements of whole brains and lateral ventricles were performed in ITK-snap. The analysis was conducted in a blinded fashion.

### Immunofluorescence

Brain sections were immunostained with the primary antibodies overnight at 4°C, followed by incubation with the secondary antibody for 2 h at room temperature. Sections were nuclei stained using Fluoroshield^™^ with DAPI (Sigma-Aldrich, F6057). Images for fluorescence quantification were obtained with an Olympus fluorescence microscope (Olympus, Tokyo, Japan) using the cellSense Software (v 1.18), while confocal microscopy (Nikon A1; Nikon Instruments Inc, NY, USA) was employed to verify antibody presence in the correct choroid plexus membrane. Images were taken with the same contrast settings and exposure times. The primary antibodies used were rabbit anti-NKCC1 (1:200, Abcam, ab59791) and rabbit anti-phosphorylated NKCC1 (pNKCC1, Thr212/Thr217, 1:200, EMD Millipore, ABS1004). The secondary antibody used was Alexa Fluor 594 donkey anti-rabbit (1:500, Invitrogen, A21207). Negative controls were made by omission of the primary antibodies and resulted in absence of any fluorescence (data not shown). Fluorescence quantification was conducted in Image J (v 1.52a) [31]. For each lateral ventricle, one image acquired at 10x magnification containing the majority of the choroid plexus was selected. The choroid plexus was outlined manually and the area and integrated density obtained. The fluorescence intensity was calculated as the integrated density divided by the area of the choroid plexus. To correct for background fluorescence, the fluorescence intensity of a region devoid of any tissue was obtained and subtracted from the fluorescence intensity of the choroid plexus. To correct for variations in stromal size, the choroid plexus stromal area was subtracted from the total choroid plexus area. The fluorescence intensity of the right and left lateral choroid plexus was averaged to give an overall fluorescence intensity for each brain section. All images were randomized and blinded prior to the analysis.

### Western blot

Choroid plexus samples were immersed in Western blot sample buffer (62.5 mmol/L Tris-HCl, pH 6.8, 2% sodium dodecyl sulfate, 10% glycerol, and 5% β-mercaptoethanol), sonicated, and the protein content determined with the Bio-Rad protein assay kit (Hercules). Then, 50 µg protein samples were loaded on a 4–20% SDS-PAGE gel. The protein samples were transferred onto a Hybond-C PURE Nitrocellulose membrane (Amersham) and blocked with 5% non-fat milk (LabScientific Inc) at room temperature overnight. After blocking, membranes were incubated with the primary antibodies overnight at 4°C. Membranes were then immunoprobed with the secondary antibodies for 1 h at room temperature. The antigen-antibody complexes were visualized with the ECL chemiluminescence system (Amersham) and exposed to a Kodak X-OMAT film. The relative densities of bands were analyzed with ImageJ (v 1.52a). The primary antibodies were rabbit anti-phosphorylated NKCC1 (pNKCC1, Thr212/Thr217, 1:200, EMD Millipore, ABS1004) and mouse β-actin (1:3333, Sigma, A5441). The second antibodies used as 1:2000 were goat-anti rabbit (Bio-Rad, 170–6515) and goat-anti mouse (Bio-Rad, 170–6516). ECL chemiluminescence system (Amersham) and Kodak X-OMAT film (Sigma) were used for final visualization. The raw western blot image is attached as Additional file 1, Fig. S2.

### ^**86**^**Rb**^**+**^**efflux assay**

Rats were anesthetized with an intraperitoneal injection of ketamine and xylazine (60 mg/ml, 6 mg/ml, 0.17 ml per 100 g body weight, ScanVet) and euthanized by decapitation. The brains were immediately isolated and immersed in ice-cold HEPES-aCSF for 10 min, followed by separation of the two hemispheres and isolation of the choroid plexus from the lateral ventricles. After acute isolation, the lateral choroid plexus was allowed to recover for 10 min in 37 °C HEPES-aCSF prior to 10 min of incubation in an isotope solution containing: ^86^Rb^+^ (1 µCi/ml, 022-105721-00321-0001, POLATOM) and ^3^H-mannitol (4 µCi/ml, NET101, Perkin Elmer). ^3^H-mannitol does not enter the choroid plexus epithelial cells and serves as an extracellular marker [32]. After isotope incubation, the choroid plexus was briefly rinsed in isotope-free 37 °C HEPES-aCSF followed by transfer into new wells containing isotope-free HEPES-aCSF at 10 s intervals for a total of 80 s. For every time point, 0.2 ml of surrounding fluid was collected and placed into scintillation vials. At the end of the experiment, the choroid plexus was placed into a scintillation vial containing 1 ml Solvable (6NE9100, Perkin Elmer) and dissolved at room temperature overnight. The isotope content was determined in 2 ml Ultima Gold™ XR scintillation liquid (6,013,119, Perkin Elmer) using the Tri-Carb 2900TR Liquid Scintillation Analyzer (Packard). For each time point, the ^86^Rb^+^ activity was corrected for extracellular background using ^3^H-mannitol. Data are shown as the natural logarithm of the ^86^Rb^+^ activity at time each point (A_T_) normalized to the initial ^86^Rb^+^ activity (A_0_) as a function of time. The slope from linear regression analysis was used to determine the ^86^Rb^+^ efflux rate constant (min^− 1^) [32, 33]. The pharmacological agent bumetanide (dissolved in DMSO to a stock concentration of 20 mM, B3023, Sigma,) was present in the efflux wells at a final concentration of 20 µM (with vehicle inclusion in the control samples).

### Ventriculo-cisternal perfusion

CSF secretion rates were quantified with the ventriculo-cisternal perfusion technique [33–36]. Anesthetized and mechanically ventilated rats were placed in a stereotactic frame, the skull exposed, and a 0.5 mm cranial burr hole drilled above the right lateral ventricle (0.3 mm posterior, 1.4 mm lateral to bregma). A 4.5 mm brain infusion cannula (Brain infusion kit2, Alzet) was inserted into the right lateral ventricle, through which pre-heated (37°C, SF-28, Warner Instruments) and gas-equilibrated aCSF containing a fluorescent dye (0.5 mg/ml tetramethylrhodamine isothiocyanate-dextran, MW = 155 kDa, T1287, Sigma) could be continuously infused at a rate of 9 µl/min using a peristaltic pump. The neck muscle layers of the animals were separated to enable fluid collection from a downstream cisterna magna puncture, which prevented the ICP elevation during the experimental procedure. Fluid was collected in 5-minute intervals over a 2-hour period by insertion of a glass capillary (30–0067, Harvard Apparatus pulled by a Brown Micropipette puller, Model P-97, Sutter Instruments) at a 5° angle into cisterna magna, through which a second glass capillary (30–0065, Harvard Apparatus) could be continuously inserted and replaced. A microplate photometer (545 nm, Synergy™ Neo2 Multi-mode Microplate Reader, BioTek Instruments) was used to measure the fluorescence content of the collected fluid samples and the CSF secretion rate was calculated from the equation:

$${V}_{p}={r}_{i}\cdot \frac{{C}_{i}-{C}_{o}}{{C}_{o}}$$

where V_p_ = CSF secretion rate (µl/min), r_i_ = infusion rate (µl/min), C_i_ = fluorescence of inflow solution (a.u.), C_o_ = fluorescence of outflow solution (a.u.), calculated based on stable time intervals from 60 to 90 min.

### Intracranial pressure and CSF outflow resistance

Anesthetized and mechanically ventilated rats were placed in a stereotactic frame and a 3.6 mm diameter cranial window was drilled with care not to damage the dura. The epidural cannula (PlasticsOne, C313G) was filled with HEPES-aCSF, connected to a pressure transducer APT300 and transducer amplifier module TAM-A (Hugo Sachs Elektronik), and secured to the skull with dental resin cement (Panavia SA Cement, Kuraray Noritake Dental Inc.). On the contralateral side of the skull, a 0.5 mm burr hole was drilled (0.3 mm posterior, 1.4 mm lateral to bregma), and a 4.5 mm brain infusion cannula (Brain infusion kit2, Alzet) was placed into the lateral ventricle. 5 µl HEPES-aCSF was injected through the epidural ICP cannula to ensure a continuous fluid column between the dura and the probe before starting the baseline ICP recording. After 20 min, heated and gas-equilibrated aCSF was infused at 5, 10, 15 and 20 µl/min for 10 min each. The CSF outflow resistance for each infusion speed was then calculated as [37]:

$${R}_{out}=\frac{{ICP}_{inf}-{ICP}_{base}}{{V}_{inf}}$$

Where ICP_inf_ is the average ICP during the last 3 min of the infusion given in mmHg, ICP_base_ is the baseline ICP in mmHg, and V_inf_ is the infusion speed in µl/min. R_out_ for each rate of infusion was calculated and averaged across all infusion rates for each rat to determine CSF outflow resistance.

### Statistics

Data analysis and statistical tests were conducted in GraphPad Prism (GraphPad Software, v 9). Data were tested for normality with the Shapiro-Wilk test. For normally distributed data, an unpaired two-tailed t-test was conducted. For non-normally distributed data, a Mann-Whitney test was conducted. For more than two groups, an ordinary one-way ANOVA followed by Sidak’s multiple comparisons test was conducted. Statistical tests are indicated in the figure legend. *P* < 0.05 was considered statistically significant. All data are presented as mean ± standard deviation and n corresponds to the number of individual animals.

## Results

### SHRs display ventricular enlargement and brain water accumulation

**Fig. 1:**
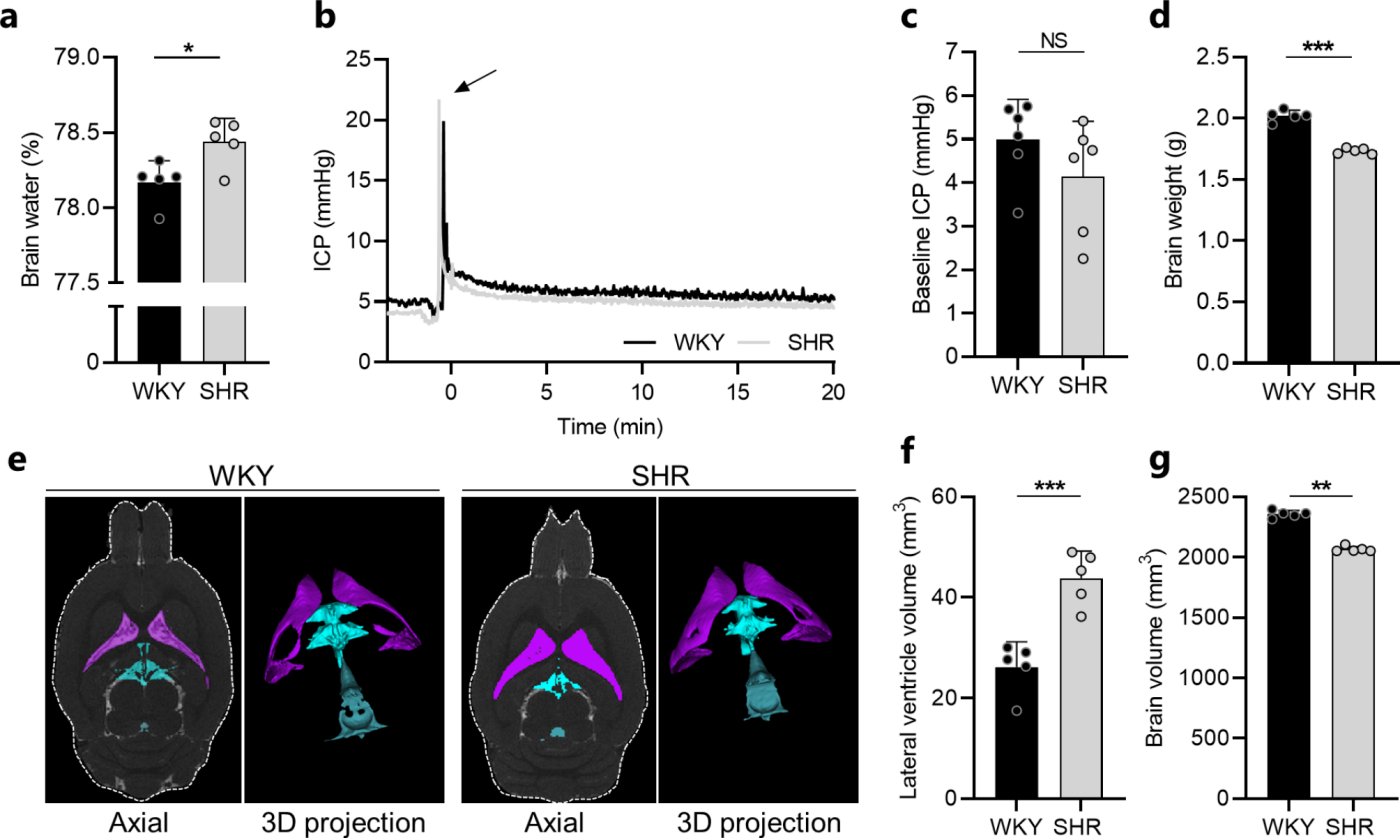
SHRs display ventricular enlargement and excessive brain water accumulation. **a** Brain water content quantified from WKY rats (n = 5) and SHRs (n = 5) using the wet and dry brain weight. **b** Representative ICP traces for one WKY rat and one SHR. Arrow indicates injection of 5 µl aCSF to assure proper ICP reading. **c** Baseline ICP in WKY rats (n = 6) and SHRs (n = 6) quantified from a stable 20 min time period. **d** Brain weight of WKY rats (n = 5) and SHRs (n = 5) assessed post mortem. **e** Representative MRI brain sections (2D axial plane and 3D projections) of one WKY rat and one SHR demonstrating the lateral ventricle volume (purple), third ventricle volume (turquoise), and fourth ventricle volume (blue). **f** Lateral ventricle volumes of WKY rats (n = 5) and SHRs (n = 5) quantified from MRI. **g** Brain volumes of WKY rats (n = 5) and SHRs (n = 5) quantified by MRI. Error bars represent standard deviation and statistical significance was tested with an unpaired two-tailed t-test or a Mann-Whitney test depending on normality. *P < 0.05, **P < 0.01, *** *P* < 0.001, NS = not significant

As SHRs are reported to develop non-obstructive hydrocephalus and accumulate CSF within their ventricular system [8–10, 13], we first quantified the brain water content of SHRs and WKY rats with the wet-dry technique [38]. The brain water content of SHRs was significantly higher than that obtained in WKY rats (3.64 ± 0.03 ml/g dry weight in SHRs vs. 3.58 ± 0.03 ml/g dry weight in WKY rats, n = 5, *P* < 0.05), which amounted to < 1% difference in brain water percentage (78.4 ± 0.2% in SHRs vs. 78.2 ± 0.1% in WKY rat, n = 5, *P* < 0.05, Fig. 1a). To determine whether the brain water accumulation in SHRs was associated with an elevated ICP as frequently observed in patients with hydrocephalus [1], an epidural ICP pressure probe was placed in a cranial window in anesthetized and mechanically ventilated SHRs and WKY rats, and the baseline ICP quantified from a stable 20 min time window (Fig. 1b). The ICP measurements, however, revealed no significant difference in baseline ICP between the two groups (4.14 ± 1.27 mmHg in SHRs vs. 4.99 ± 0.92 mmHg in WKY rats, n = 6, *P* = 0.21, Fig. 1c). Hence, the brain water accumulation observed in SHRs did not translate to an elevated ICP. As the brain is confined within the rigid skull, the Monro-Kellie doctrine [39] dictates that changes in the volume of one brain compartment (CSF, blood, brain parenchyma) must be compensated by changes in another brain compartment to avoid changes in ICP. Accordingly, the brain mass of SHRs was significantly smaller than that observed in the WKY rats (1.73 ± 0.02 g in SHRs vs. 2.02 ± 0.05 g in WKY rats, n = 5, *P* < 0.001, Fig. 1d). Taken together, SHRs display brain water accumulation, which may occur at the expense of brain parenchymal volume.

To characterize the distribution of the excessive brain water, SHRs and WKY rats were subjected to MRI, which allowed for quantification of distinct CSF volume compartments (Fig. 1e). MRI revealed significant enlargement of the lateral ventricles in SHRs to nearly twice the size of that in WKY rats (43.8 ± 5.3 mm^3^ in SHRs vs. 26.1 ± 5.0 mm^3^ in WKY rats, n = 5, *P* < 0.001, Fig. 1f). None of the remaining CSF volumes quantified (third ventricle, fourth ventricle, other CSF) were enlarged in SHRs (Fig. S1, Additional file 1). In agreement with the postmortem assessments of brain mass, the brain volume of SHRs quantified by MRI was significantly smaller than that of WKY rats (2066 ± 22 mm^3^ in SHRs vs. 2357 ± 30 mm^3^ in WKY rats, n = 5, *P* < 0.01, Fig. 1g). The CSF accumulation in SHRs appeared not to originate from changes in blood gas or electrolyte content, which were similar in SHRs and WKY rats, with the exception of lower blood glucose levels in SHRs (see Table S1, Additional file 2). Taken together, SHRs display ventricular enlargement and brain water accumulation characteristic of that observed in human patients with hydrocephalus.

### SHRs demonstrate choroid plexus transporter alterations

**Fig. 2:**
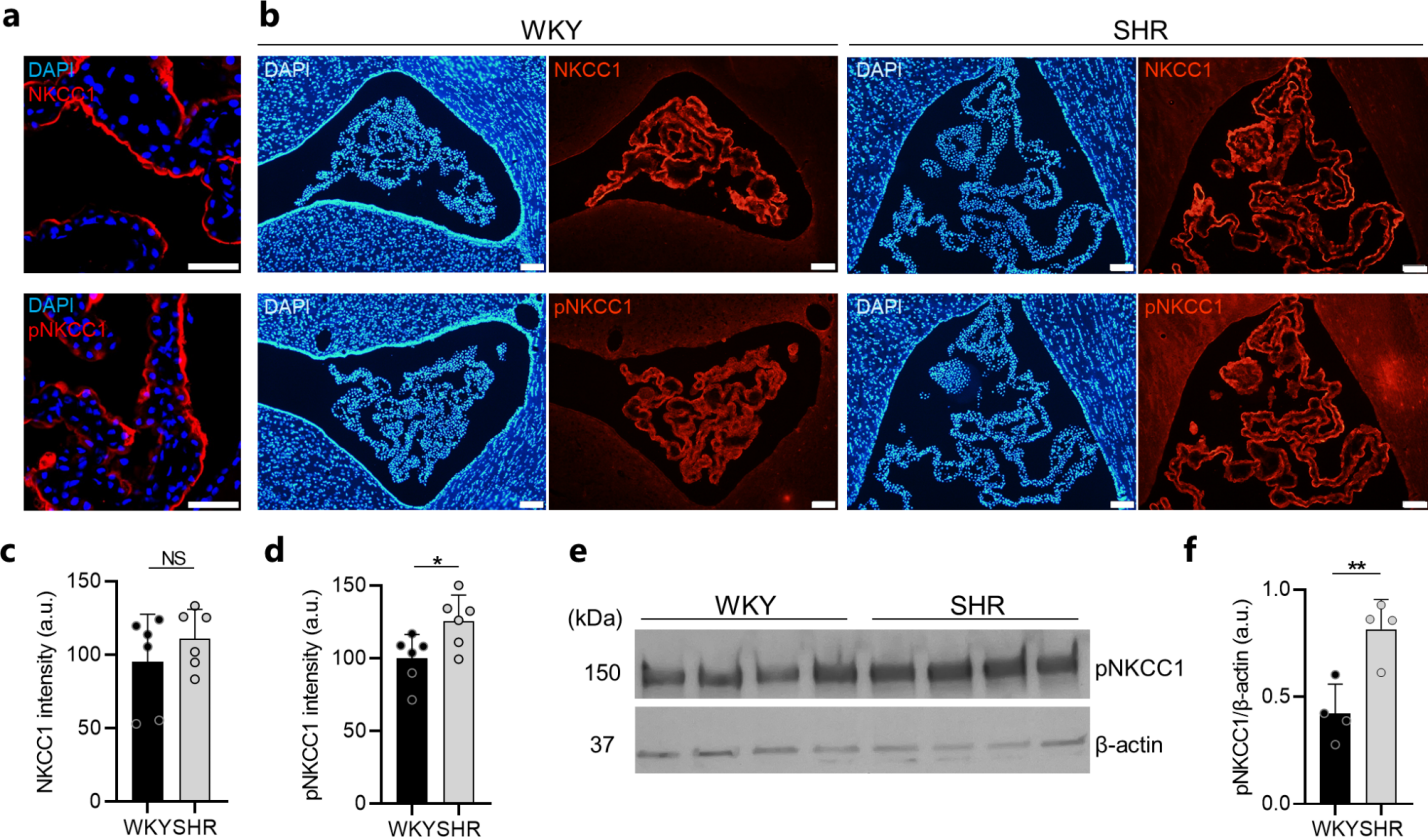
The SHR choroid plexus demonstrates upregulation of pNKCC1. **a** Confocal microscopy images demonstrating apical localization of NKCC1 (red, top panel) and pNKCC1 (red, lower panel) in SHR choroid plexus. Cell nuclei are stained with DAPI (blue). Scale bar: 50 μm. **b** Representative immunofluorescence images of the choroid plexus from WKY rats (left panels) and SHRs (right panels) stained for NKCC1 (red, top panels), pNKCC1 (red, lower panels), and cell nuclei (DAPI, blue). Scale bar: 100 μm. **c-d** Quantification of NKCC1 (**c**) or pNKCC1 (**d**) fluorescence intensity in choroid plexus from WKY rats and SHRs, n = 6 in each group. **e** Western blot of pNKCC1 in choroid plexus from WKY rats and SHRs. β-actin served as loading control. **f** quantification of pNKCC1 abundance in choroid plexus from WKY rats (n = 4) and SHRs (n = 4) normalized to β-actin. Error bars represent standard deviation and statistical significance was tested with an unpaired two-tailed t-test or a Mann-Whitney test. * *P* < 0.05, **P < 0.01, NS = not significant

The CSF is predominantly produced by the secretory actions of the choroid plexus residing in each of the four brain ventricles [40]. Although the exact molecular mechanisms underlying choroid plexus CSF secretion remain unresolved, the water-translocating Na^+^/K^+^/2Cl^−^ cotransporter NKCC1 has been identified as one key contributor [33, 36, 41]. To elucidate whether the ventricular enlargement and brain water accumulation observed in SHRs was associated with NKCC1 alterations, the choroid plexus from SHRs and WKY rats was immunostained for NKCC1 and pNKCC1, the phosphorylated, activated form of NKCC1, which both localize to the luminal (apical) membrane of the choroid plexus epithelium (Fig. 2a). Quantification of fluorescence intensity (Fig. 2b) revealed unaltered NKCC1 expression (110 ± 20 a.u. in SHRs vs. 95 ± 32 a.u. in WKY rats, n = 6, *P* = 0.39, Fig. 2c) but significant increase of pNKCC1 in choroid plexus from SHRs when compared to WKY rats (126 ± 18 a.u. in SHRs vs. 100 ± 17 a.u. in WKY rats, n = 6, *P* < 0.05, Fig. 2d). To verify the pNKCC1 increase in SHRs, western blotting was employed (Fig. 2e), which revealed an almost twofold increase of pNKCC1 in the choroid plexus from SHRs when compared to that of WKY rats (0.82 ± 0.14 a.u. in SHRs vs. 0.42 ± 0.14 a.u. in WKY rats, n = 4, P < 0.01, Fig. 2f). Taken together, the SHR choroid plexus demonstrates increased levels of pNKCC1, the phosphorylated, activated form of NKCC1.

**Fig. 3:**
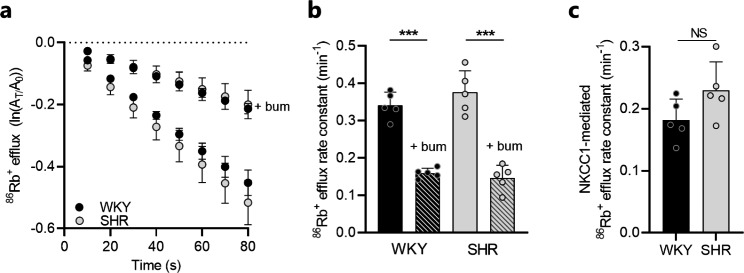
Choroid plexus NKCC1 transport activity is unaltered in SHRs. **a** Loss of ^86^Rb^+^ from the choroid plexus as a function of time in WKY rats (n = 5) and SHRs (n = 5) in presence or absence of 20 µM bumetanide (bum). The y-axis is the natural logarithm of the choroid plexus ^86^Rb^+^ amount left at time T (A_T_) divided by the initial amount at time 0 (A_0_). **b** Efflux rate constant for ^86^Rb^+^ in WKY rats (n = 5) and SHRs (n = 5) in presence or absence of 20 µM bumetanide (bum). **c** NKCC1-mediated ^86^Rb^+^ efflux rate constant (bumetanide-sensitive fractions) in WKY rats (n = 5) and SHRs (n = 5). Error bars represent standard deviation and statistical significance was tested with one-way ANOVA followed by Sidak’s multiple comparisons test or an unpaired-two tailed t-test. ***P < 0.001, NS = not significant

To determine whether the increased expression of pNKCC1 in the SHR choroid plexus translated to an increased NKCC1 transport activity, the transport rate of NKCC1 was quantified with an ex vivo radio-isotope based transport assay in choroid plexus acutely isolated from SHRs and WKY rats. In this assay, radioactive ^86^Rb^+^ was employed to mimic the movement of K^+^ through NKCC1 to enable assessment of the NKCC1 transport activity. The outward transport of ^86^Rb^+^ by NKCC1 was monitored as choroid plexus ^86^Rb^+^ efflux as a function of time (Fig. 3a). The ^86^Rb^+^ efflux rate was diminished by ~ 50–60% in presence of the NKCC1 inhibitor bumetanide in both SHRs and WKY rats (SHR: 0.38 ± 0.06 min^− 1^ vs. 0.15 ± 0.03 min^− 1^; WKY: 0.34 ± 0.04 min^− 1^ vs. 0.16 ± 0.01 min^− 1^, n = 5 of each, *P* < 0.001, Fig. 3b). Although there was a tendency towards an increased NKCC1-mediated ^86^Rb^+^ efflux rate (the bumetanide-sensitive fraction) in SHRs when compared to that of WKY rats, the difference was not significant (0.23 ± 0.05 min^− 1^ in SHRs vs. 0.18 ± 0.03 min^− 1^ in WKY rats, n = 5, *P* = 0.10, Fig. 3c). Collectively, the above data indicate that the SHR choroid plexus displays subtle alterations in NKCC1 activation, which could contribute to altered CSF dynamics in these rats.

### CSF secretion rate and CSF outflow resistance are not elevated in SHRs

**Fig. 4:**
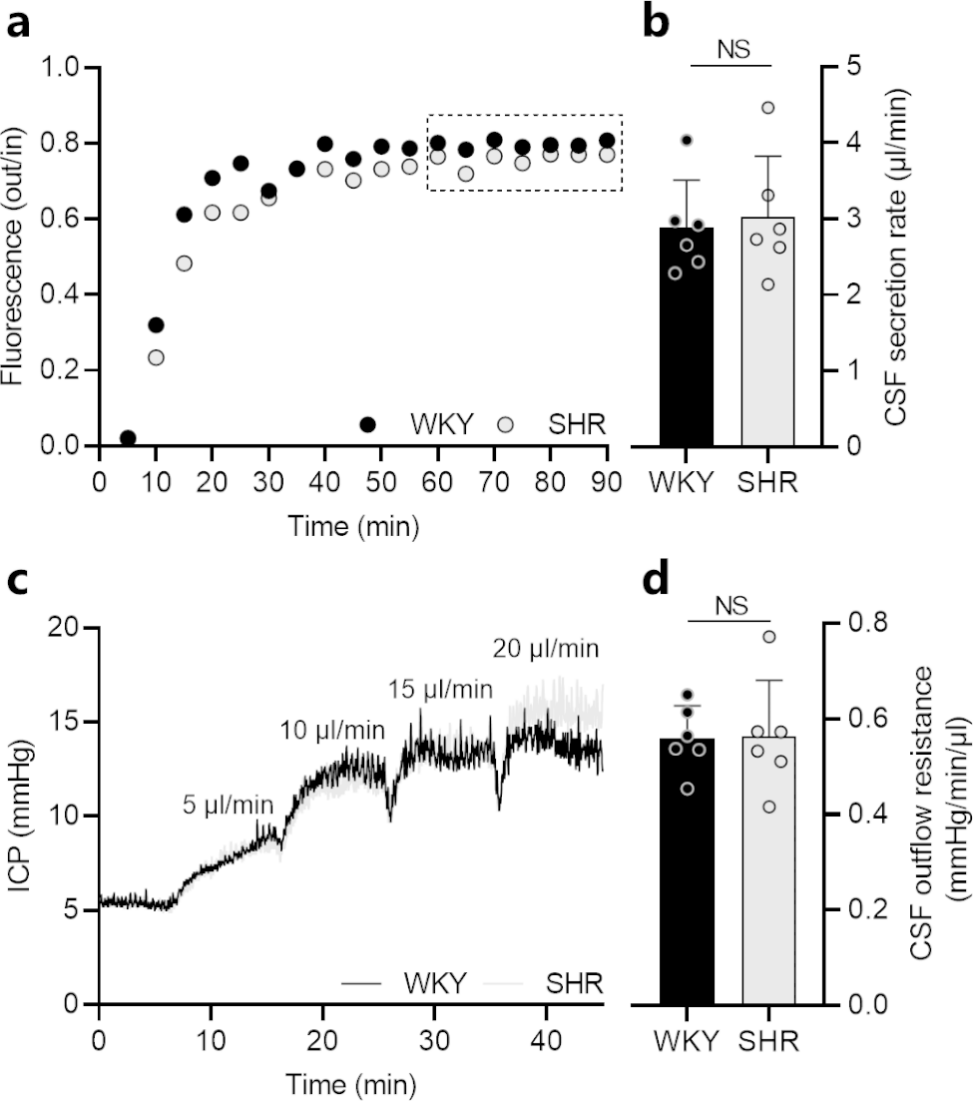
The CSF secretion rate and outflow resistance is not elevated in SHRs. **a** Representative time course traces of the fluorescence ratio of dextran (outflow/inflow) during ventriculo-cisternal perfusion of one WKY rat and one SHR. Squared insert indicates the region used for quantification of CSF secretion rates. **b** CSF secretion rate in WKY rats (n = 6) and SHRs (n = 6) quantified from the fluorescence ratio of dextran. **c** Representative ICP traces of one WKY rat and one SHR undergoing measurements of CSF outflow resistance by infusion of aCSF at rates indicated in the figure. **e** CSF outflow resistance in WKY rats (n = 6) and SHRs (n = 6). Error bars represent standard deviation and statistical significance was tested with an unpaired two-tailed t-test. NS = not significant

To resolve whether the ventricular enlargement and excessive brain water accumulation observed in SHRs originated from an increased CSF secretion, possibly mediated by the subtle alterations in NKCC1 activation, we quantified the CSF secretion rate in anesthetized and mechanically ventilated SHRs and WKY rats with the ventriculo-cisternal perfusion technique [33–36]. Here, heated and gas-equilibrated aCSF containing a fluorescent dye (dextran) was perfused at a constant rate into the lateral ventricle of the rats and CSF samples were continuously collected from a downstream cisterna magna puncture. The CSF secretion rates were quantified based on the fluorescence content obtained from a stable 30 min time window (Fig. 4a). Analysis of the fluorescence content revealed similar CSF secretion rates in SHRs and WKY rats (3.02 ± 0.80 µl/min in SHRs vs. 2.88 ± 0.62 µl/min in WKY rats, n = 6, *P* = 0.75, Fig. 4b). The ventricular enlargement and excessive brain water accumulation observed in SHRs thus appeared not to originate from increased CSF secretion. These pathological alterations could, alternatively, originate from inefficient CSF drainage, thereby promoting accumulation of CSF. To elucidate the CSF drainage capacity, we determined the CSF outflow resistance [37] in anesthetized and mechanically ventilated SHRs and WKY rats. In brief, heated and gas-equilibrated aCSF was infused into the right lateral ventricle at different rates with continuous ICP monitoring (Fig. 4c), which allowed for determination of the CSF outflow resistance. However, the CSF outflow resistance did not differ significantly in SHRs and WKY rats (0.56 ± 0.12 mmHg/min/µl in SHRs vs. 0.56 ± 0.07 mmHg/min/µl in WKY rats, n = 6, *P* = 0.95, Fig. 4d). The CSF drainage efficiency thus appeared intact in SHRs. Taken together, neither increased CSF secretion nor inefficient CSF drainage appear to underline the ventriculomegaly and excessive brain water content observed in SHRs.

## Discussion

Here, we demonstrate that development of hydrocephalus in SHRs is associated with enlargement of the lateral ventricles, brain water accumulation, and choroid plexus transporter alterations. However, we found no evidence of altered CSF dynamics as evident by similar CSF production rates, ICPs, and CSF outflow resistances in SHRs and their normotensive control counterparts, the WKY rats. The pathophysiological mechanism underlying development of hydrocephalus in SHRs thus remains unresolved.

Progressive ventricular enlargement in SHRs has been documented since the 1980s [8, 9] with an apparent onset around seven weeks of age [10, 11]. In agreement, we observed significant enlargement of the lateral ventricles in nine-week-old SHRs. However, none of the remaining CSF volumes quantified (third ventricle, fourth ventricle, other CSF) were enlarged, which may be ascribed to their young age, as CSF volumes in these rats are reported to increase with age, reflected by significant enlargement of not only the lateral ventricles, but also the third ventricle, cisterna magna, and total CSF volume in aged SHRs [12, 13]. The ventricular enlargement in SHRs was accompanied by an elevated brain water content, as observed by others [13], indicative of CSF accumulation.

The CSF accumulation, however, did not translate to an elevated ICP, as quantified through an epidural pressure probe. This finding is in agreement with previous reports [9, 42], in which ICP was measured by pressure probes residing in cisterna magna [42] or in the ventricles [9]. One study contradicts this finding and observed elevated ICP in SHRs [43]. ICP is reported to depend on breathing parameters [44], and SHRs present with compensated respiratory alkalosis due to chronic hyperventilation, even before development of hypertension and ventricular enlargement [45]. Hyperventilation in SHRs could potentially mask the effects of factors that would increase ICP by reducing blood pCO_2_ levels and cerebral blood volume. Therefore, in this study, the rats were mechanically ventilated during the ICP recordings to overcome potential confounding factors related to uncontrolled breathing. Different methodological approaches thus demonstrate that CSF accumulation in SHRs does not elevate the ICP. Although the CSF accumulation observed in patients with hydrocephalus often translates to a clinically elevated ICP [1], these two parameters are not always correlated in humans either, as exemplified by patients with idiopathic intracranial hypertension, in whom the ICP is elevated despite lack of ventriculomegaly [46] and by patients with normal pressure hydrocephalus, in whom the ICP remains unaltered, or only slightly elevated, despite excessive CSF accumulation [47]. The latter pattern resembles that of the SHR, which, in comparison to the WKY rats, additionally display faster age-dependent decline of cognition and hyperactive bladders [14, 48–50], two prominent features of Hakim’s triad of symptoms characterizing normal pressure hydrocephalus [51]. To our knowledge, the last feature of Hakim’s triad; gait disturbances, has yet to be reported for the SHR rats for them to fully encompass all symptoms of normal pressure hydrocephalus. The unaltered ICP in SHRs could possibly originate from a reduction in brain parenchymal volume as evident from their smaller brain volumes in comparison to WKY rats, observed in this study and previously by others [12–14]. The reduction in brain parenchymal volume may serve to homeostatically compensate for the accumulation of CSF in accordance with the Monro-Kellie doctrine [39]. However, whether the reduction in brain parenchymal volume arises as a consequence of loss in microvascular tissue and neurons causing cerebral atrophy [14, 52, 53], thereby passively allowing the expanding ventricles to fill with CSF, or whether the CSF accumulation actively distends the ventricles and compresses the surrounding brain parenchyma remains unresolved.

CSF accumulation might arise from increased CSF secretion. However, no evidence of increased CSF secretion in SHRs was found in the present study, albeit increased levels of phosphorylated NKCC1 were observed in the SHR choroid plexus. Water translocation through NKCC1 normally constitutes ~ 50% of the total CSF secretion in mice, rats, and dogs [33, 36, 41] and increased phosphorylation of NKCC1 has previously been associated with increased CSF secretion in rodent models of posthemorrhagic hydrocephalus [21, 23] and postinfectious hydrocephalus [19]. However, as the CSF secretion rate appeared similar in SHRs and WKY rats, the physiological importance of increased phosphorylated NKCC1 in the SHR choroid plexus remains unclear. The present finding of unaltered CSF secretion in SHRs aligns well with some earlier studies [42, 54] but contradicts others, which demonstrate increased CSF secretion in SHRs when compared to WKY rats [11, 43, 55]. The studies that demonstrated increased CSF secretion in SHRs employed the ventriculo-cisternal perfusion technique [43, 55] or the direct method [11] for estimation of CSF secretion rates, while the studies that found no evidence of increased CSF secretion assessed the amount of CSF collected over a certain period of time [54] or calculated the rate of CSF refilling after CSF withdrawal [42]. The present study employed the ventriculo-cisternal perfusion technique to evaluate CSF secretion rates, but in contrast to the remaining studies, the rats were mechanically ventilated during the experimental procedure to avoid the artificial lowering of CSF secretion rates associated with absence of mechanical ventilation [33]. Of note, a recent study employed non-invasive arterial spin labeling MRI to quantify the amount of water delivered from the choroid plexus blood circulation to the ventricles as a proxy for CSF secretion and observed significantly less water transport across the choroid plexus of SHRs when compared to WKY rats, indicative of reduced CSF secretion [56]. Hence, whether the CSF secretion is altered in SHRs remains controversial, and this may, at least in part, be related to use of different experimental measurement techniques. Presently, we cannot exclude that a subtle increase in CSF secretion that is too small to be detected with the ventriculo-cisternal perfusion technique may gradually, over time, contribute to the observed ventriculomegaly in SHRs.

The CSF accumulation observed in SHRs could, alternatively, originate from inefficient CSF drainage, thereby promoting accumulation of CSF. To the best of our knowledge, the present study is the first to quantify the CSF outflow resistance in SHRs in an attempt to uncover whether inefficient CSF drainage could underlie development of hydrocephalus in these rats. However, we observed similar CSF outflow resistances in SHRs and WKY rats, which implies no drainage deficiency in SHRs. With the undisturbed CSF secretion rate and CSF drainage capacity, the underlying molecular mechanisms supporting the SHR ventriculomegaly remain unresolved.

Analysis of blood gas and electrolyte content in SHRs and WKY rats provided no further clues to the disease pathogenesis, except for lower blood glucose levels in SHRs, which could be indicative of an altered metabolic activity in these rats. Notably, all experiments in the present study were conducted using nine-week-old rats, an age at which hydrocephalus already appears pronounced in SHRs. The pathogenic alterations underlying development of hydrocephalus in SHRs could potentially take place at an earlier age, possibly even prior to the onset of hydrocephalus. Hence, we cannot rule out that the CSF secretion rate or the CSF outflow resistance may be altered at an earlier age in SHRs, which may contribute to the disease pathogenesis. Hydrocephalus development in SHRs may, alternatively, be genetically determined as pharmacologically induced reduction of the SHR blood pressure fails to attenuate the observed ventriculomegaly [9], or it may arise from pathological alterations not investigated in the current study. Amongst other pathological alterations related to brain fluid management, SHRs are reported to display enhanced interstitial fluid drainage towards the ventricular system [57], an altered distribution of brain and choroid plexus aquaporins [58, 59], altered diffusional properties in various brain regions [13], and impaired glymphatic transport [12]. Hydrocephalus development in SHRs has, moreover, been linked to choroid plexus injury, ventricular wall damage, and activation of epiplexus macrophages, which may be ameliorated by treatment with anti-inflammatory minocycline, suggesting a possible inflammatory component to the disease pathogenesis [10]. Inflammatory events at the choroid plexus may lead to increased NKCC1 phosphorylation [19, 21, 23] and may contribute to the increased phosphorylation described in the current study. However, overall, the exact pathological mechanisms underlying development of hydrocephalus in SHRs await determination.

In conclusion, SHRs display ventricular enlargement and brain water accumulation with a striking absence of ICP elevation, possibly due to compensatory brain tissue volume reduction. The increased brain water content did not arise through increased CSF secretion nor through inefficient CSF drainage, and its origin therefore remains unresolved. Future revelation of the molecular mechanisms underlying SHR ventriculomegaly could aid in unravelling the etiology of pathologies such as normal pressure hydrocephalus, in which patients also display ventriculomegaly with no ICP elevation. Despite their normal ICP, these patients often benefit from ventricular shunt placement [60–62], suggesting that the increased brain fluid content influences the pathology.

## Electronic supplementary material

Below is the link to the electronic supplementary material.

Additional File 1: CSF volumes, western blot

Additional File 2: Blood gas analysis

## Data Availability

The datasets used and/or analyzed during the current study are available from the corresponding author on reasonable request.

## Abbreviations

aCSF: artificial CSF
CSF: Cerebrospinal fluid
ICP: Intracranial pressure
MRI: Magnetic resonance imaging
NKCC1: Na^+^/K^+^/2Cl^−^ cotransporter
SHR: Spontaneously hypertensive rat
WKY: Wistar-Kyoto
