# Supplementary material for: Spontaneously hypertensive rats can become hydrocephalic despite undisturbed secretion and drainage of cerebrospinal fluid
Source: Fluids Barriers CNS. 2023 Jul 4;20:53. doi: 10.1186/s12987-023-00448-x (PMC10318838; doi:10.1186/s12987-023-00448-x)
Supplement: Supplementary file 1 — Additional File 1: CSF volumes, western blot [file 12987_2023_448_MOESM1_ESM.pdf]

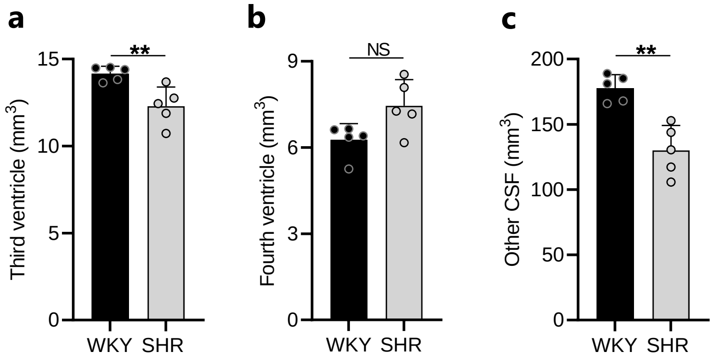

Fig. S1. CSF volumes in SHRs and WKY rats. **a** Third ventricle volumes in WKY rats (14.2 ± 0.4 mm^3, n = 5) and SHRs (12.3 ± 1.1 mm^3, n = 5) quantified by MRI. **b** Fourth ventricle volumes in WKY rats (6.3 ± 0.6 mm^3, n = 5) and SHRs (7.5 ± 0.9 mm^3, n = 5) quantified by MRI. **c** Other CSF volumes in WKY rats (178 ± 10 mm^3, n = 5) and SHRs (130 ± 19 mm^3, n = 5) quantified by MRI. Data are presented as mean ± standard deviation and statistical significance was tested with an unpaired two-tailed t-test or a Mann-Whitney test depending on normality. ** $P < 0.01$. NS = not significant.

Fig. S1

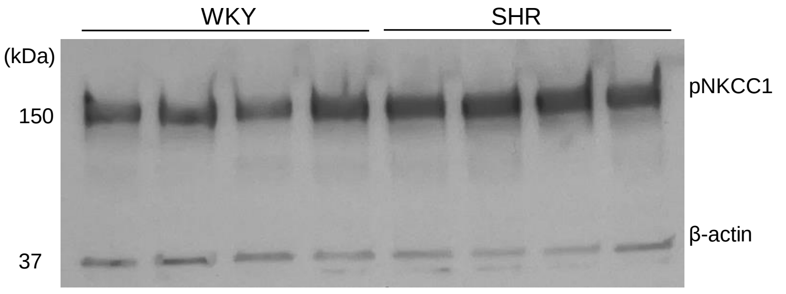

Fig. S2. Western blot. Raw western blot of choroid plexus acutely isolated from WKY rats (n = 4) and SHRs (n = 4) stained with anti-pNKCC1 and anti β-actin antibodies.

Fig. S2
