# Supplementary material for: Spontaneously hypertensive rats can become hydrocephalic despite undisturbed secretion and drainage of cerebrospinal fluid
Source: Fluids Barriers CNS. 2023 Jul 4;20:53. doi: 10.1186/s12987-023-00448-x (PMC10318838; doi:10.1186/s12987-023-00448-x)
Supplement: Supplementary file 2 — Additional File 2: Blood gas analysis [file 12987_2023_448_MOESM2_ESM.docx]

**Supplementary Table S1** **Blood gas analysis in anesthetized and mechanically ventilated SHRs and WKY rats.**

|  | **WKY** | | | **SHR** | | |  |
| --- | --- | --- | --- | --- | --- | --- | --- |
|  | mean | SD | n | mean | SD | n | ***P*-value** |
| **Blood gases** |  | | | | | | |
| pCO_2_ (kPa) | 4.44 | 0.52 | 5 | 4.43 | 0.23 | 5 | 0.98 |
| pO_2_ (kPa) | 19.5 | 1.4 | 5 | 18.3 | 2.0 | 5 | 0.30 |
| **Electrolytes** |  | | | | | | |
| K^+^ (mM) | 5.06 | 0.51 | 5 | 4.56 | 0.36 | 5 | 0.11 |
| Na^+^ (mM) | 141 | 2 | 5 | 141 | 3 | 5 | 0.78 |
| Ca^2+^ (mM) | 0.86 | 0.03 | 5 | 0.85 | 0.07 | 5 | 0.71 |
| Cl^-^ (mM) | 104 | 2 | 5 | 106 | 2 | 5 | 0.16 |
| HCO_3_^-^ (mM) | 22.6 | 1.1 | 5 | 22.8 | 1.0 | 5 | 0.72 |
| **Metabolites** |  | | | | | | |
| cGlu (mM) | 28.4 | 1.8 | 5 | 22.3 | 3.3 | 5 | **<0.01** |
| cLac (mM) | 0.88 | 0.11 | 5 | 1.04 | 0.21 | 5 | 0.17 |
| **Acid-base status** |  | | | | | | |
| pH | 7.42 | 0.03 | 5 | 7.42 | 0.02 | 5 | 0.81 |

Data were analysed with an unpaired two-tailed t-test. Significant *P*-values (*P*<0.05) are highlighted in bold.
